# Property Relationship in Organosilanes and Nanotubes Filled Polypropylene Hybrid Composites

**DOI:** 10.3390/ma7107073

**Published:** 2014-10-20

**Authors:** Alejandra J. Monsiváis-Barrón, Jaime Bonilla-Rios, Antonio Sánchez-Fernández

**Affiliations:** Tecnológico de Monterrey Av. Eugenio Garza Sada Sur 2501, Tecnológico, 64849 Monterrey, Nuevo León, Mexico

**Keywords:** organosilanes, silanol, halloysite nanotubes, carbon nanotubes, polypropylene, sol-gel process, melt-blending

## Abstract

Polypropylene composites with different filler contents were prepared by creating a masterbatch containing 3 wt%. filler. A variety of silanol groups were used to synthetized three compounds in different media trough a sol-gel process with acetic acid, formic acid and ammonium hydroxide as catalysts. Besides, four different nanotubular fillers were also used to analyze their behavior and compare it with the effect caused by the silanol groups. These tubular structures comprise: unmodified halloysite, carbon nanotubes and functionalized halloysite and carbon nanotubes. Morphological characterization in SEM and STEM/TEM showed dispersion in the polypropylene matrix. According to TGA and DSC measurements thermal behavior remain similar for all the composites. Mechanical test in tension demonstrate that modulus of the composites increases for all samples with a major impact for materials containing silanol groups synthetized in formic acid. Rheological measurements show a significantly increment in viscosity for samples containing unmodified and modified carbon nanotubes. No difference was found for samples containing silanol groups and halloysite when compared to neat polypropylene. Finally, the oxygen transmission rate increased for all samples showing high barrier properties only for samples containing natural and functionalized halloysite nanotubes.

## 1. Introduction

The incorporation of inorganic particulate fillers has been proved to be an effective way for the improvement and enhancement in properties, and in particular the toughness and thermal, of polymeric materials [1]. Polypropylene (PP) filled with particulate fillers has been widely studied and presently the research of this polymeric matrix is still increasing. The versatility of PP and its good processability characteristics have also made it favorable for research and development [2]. That means a composite with improved properties and lower particle concentration is highly desired. As it was mentioned in a previous work [3] two of the most important characteristics to produce a composite with good properties remain in the ability of the polymeric matrix and the particle to have enough interfacial adhesion as well as in achieving an homogeneous dispersion of the particles within the matrix; this statement is proved and supported for most of the literature already published [2,4,5]. However, good dispersion of filler remains the biggest challenge for researchers because of the tendency that particles have to form agglomerates. Consequently, the so-called nanoparticle filled polymers contain a number of clusters of particles and may therefore exhibit properties even worse than conventional particle/polymer systems. It has been documented that the typical filler contents for succeeding a good enhancement of performance is as high as 20% by volume [1,6]; however when more nanoparticles are added, the tensile yield strength becomes lower due to an increased probability of breaking and splitting of the agglomerated nanoparticles [7,8]. Also, such kinds of nanocomposites do not resist crack propagation under higher speed load as effectively as matrix resins, thus exhibiting reduced impact strength values [9]. Based on these results, it can be inferred that it might be impossible to pursue a nano-scale dispersion of the particles, especially when considering the high viscosity of the polymer melt as well as the poor interaction between the hydrophilic fillers and the hydrophobic matrix. Besides the dispersion status promotion, a lot of emphasis should be focused on the modification of the agglomerates themselves by means of a special surface treatment of the nanoparticles as stated by Rong *et al*. [10].

In this paper, a combination of *in situ* polymerization and melt-compounding is developed to overcome the challenges that most researchers experience. In the first stage sol-gel process is used to obtain organosilanes where polymers can be kinetically trapped within inorganic matrices under the right set of conditions prior to significant phase separation. The second stage comprises the mechanical mixing of synthetized silanols and nanotubular structures with the polymer to compare the different materials obtained and how properties changed depending on the filler added. The purpose of this paper is to show how efficiently the mechanical, morphological, barrier and thermal properties can be improved by the approach described. PP was chosen as the matrix and three organosilanes were synthetized as fillers to compare their effect against three different nanotubular structures: unmodified halloysite (HNT), carbon nanotubes (CNT) and functionalized carbon nanotubes (CNTF).

## 2. Experimental Section

### 2.1. Materials

As matrix, a clarified random copolymer of polypropylene (PP) Extrusion–Blown grade was used. This thermoplastic satisfies FDA requirements according the specifications mentioned in 21 CFR 177.1520 (Pro-fax SL262MW, Indelpro S.A de C.V, Tamaulipas, Mexico) with a melt flow index of 1.8 dg/min and a density of 0.9 g/cm^3^. Several approaches were developed in order to test the effect of the HNT, the CNT untreated and treated and the three different products synthetized containing organosilane molecules.

### 2.2. Synthesis of Organosilanes

Trimethoxy(propy)silane (TMOPS, 97%) was purchased from Sigma Aldrich and used without further purification. Distillated water and propyl alcohol were used for the preparation of solutions. Acetic acid, formic acid and ammonium hydroxide are the three different environments were reactions were carried out respectively.

Three different organosilanes samples were obtained by sol-gel process. 20 mL of isopropyl alcohol and 250 mL of distillated water were placed in a glass container with the reagents, 50 mL of TMOPS and 12.5 mL of the different media as shown in Table 1. The mixture was stirred at low velocity and 50 °C for 2 h. After completion of reactions a settling time of 48 h was fixed for the diluent to evaporate and phase separation was observed. Decantation and distillated water washing steps were followed afterwards; finally, another 48 h of resting time was necessary to let the rest of solvent and water evaporate.

**Table 1:** Concentrations used for the synthesis of organosilanes samples trough sol-gel process.

Reaction media (12.5 mL)	Distillated water (mL)	Isopropyl alcohol (mL)	TMOPS (mL)
Acetic acid (C_2_H_4_O_2_)	250	20	50
Formic acid (C_3_H_4_O_3_)			
Ammonium hydroxide (NH_4_OH)			

### 2.3. Nanotubes

Four different nanotubes structures were used for this research. The first and second nanotubes are Natural halloysite (Kaolin clay) purchased from Sigma Aldrich with dimensions (*d* × *L*) 30–70 nm × 1–3 μm, surface area of 64 m^2^/g and density of 2.53 g/cm^3^ and treated halloysite nanotubes with poly (meth acrylic acid). The third and fourth nanotubes were untreated and treated carbon nanotubes all of them multiwall carbon nanotubes (MWCNTs) (CM-95), purchased from Aldrich, prepared by a chemical vapor deposition method. The range in diameter was 6–13 nm; the length of the tubes was 5 μm; and the purity was greater than 95%. Functionalization of the treated carbon nanotubes is fully described by Sánchez-Fernández in a previous work already published [11]. Thriethoxy(octyl)silane with a purity of 97.5% (Aldrich) and 3-glycidoxypropyltrimethoxysilane with a purity of 98% (Aldrich) were used as the silane functionalization agents. The reagents used for the acid treatment were nitric acid (70%, Aldrich), sulfuric acid (95%–98%, Aldrich), acetone (99.5%, Aldrich), and ethanol (99.5%, Aldrich).

### 2.4. Sample Preparation

PP composites based on silanol, halloysite nanotubes and carbon nanotubes were fabricated via melt–mixing, using a 3 head mixer (ATR Plati-Corder BRABENDER®) at 190 °C with a speed of 10 rpm for 5 min set as feeding time and 50 rpm for 15 min. These conditions were selected from an optimization process already developed by Bonilla *et al*. [12], where these conditions resulted as the best to process PP matrix.

The mass fraction of filler within the polymeric matrix was kept constant at 3 wt%. and no other additive was used in the preparation of samples. The prepared materials were press-compressed at 190 °C and at 15 MPa for 5 min followed by cooling at room temperature. Finally, samples were shaped into the desired size for further measurements. Code names for each composite/blend are listed in Table 2.

**Table 2:** Nomenclature for sample prepared in a 3-head mixer.

Matrix	Filler	Code name
PP-SL262MW	–	PP
PP-SL262MW	Sol-gel (catalyst: acetic acid)	PP-Acetic
PP-SL262MW	Sol-gel (catalyst: formic acid)	PP-Formic
PP-SL262MW	Sol-gel (catalyst: ammonium hydroxide)	PP-Base
PP-SL262MW	Natural HNT	PP-HNT
PP-SL262MW	Functionalized HNT	PP-PolyHNT
PP-SL262MW	Untreated CNT	PP-CNT
PP-SL262MW	Functionalized CNT	PP-CNTF

### 2.5. Thermal Analysis

The measurements were carried out in a thermogravimetric-analyzer (TGA) coupled with DSC from TA Instruments (STD Q600, New Castle, DE, USA) to identify any changes in the thermal properties, crystallization phenomenon as well as degradation of the sample due to filler presence. This was achieved by measuring the temperature and the difference of temperature of the sample and a thermally inert reference during simultaneous heating or cooling under identical conditions. Procedure included a heat-cool-reheat cycle from 10 °C to 800 °C with a temperature rate of 10 °C/min. The first heating cycle serves to remove previous thermal history from the sample.

The sample was calibrated with Indium metal according to procedure ASTM E794 “Melting Crystallization Temperatures by Thermal Analysis” [13].

### 2.6. Rheological Testing

Frequency sweeps were included in order to characterize the rheological behavior of samples with different fillers in PP matrix. The preparation of samples for the rheological measurements was performed through press compression (25 tons at 190 °C, for 5 min) in a CARVER 4122 (Wabash, IN, USA) heating press and round samples with a diameter of 25 mm and a width of 1 mm were obtained.

#### Frequency Sweeps

In rotational methods the test fluid is continuously sheared between two surfaces, one or both of which are rotating. These devices have the advantage of being able to shear the sample for an unlimited period of time under controlled rheometric conditions, incorporating oscillatory or normal stress tests. An Anton Paar Physica MCR301 rheometer (Houston, TX, USA) was used to run frequency sweeps from 1000 to 0.01 rad·s^−1^. 10% strain and temperatures of 170 °C, 180 °C and 190 °C were included in the set parameters. Configuration was selected as 25 mm parallel plate geometry and 1 mm gap. Data obtained at different temperatures was brought into a single master curve according to Okamoto *et al.* [14].

### 2.7. Morphological Characterization

Sample cutting was carried out in an ultra-microtome Powertome XL (RMC) (Tucson, AZ, USA). A glass knife was used for the thick a face cut and afterwards the cutting in the next section to obtain a smoothed surface a diamond knife was used. The sections were floated in de-ionized water to select and place them in copper grids supports.

#### 2.7.1. SEM

In order to observe the particles dispersion in the different prepared materials, SEM images were taken using a microscope SEM-FEI Nova NanoSEM 200 (Hillsboro, TX, USA) with an acceleration voltage of 12 kV and a low vacuum detector. The EDS elemental analysis was carried out with an INCA-x-sight. The samples analyzed by SEM include those with nanotubular fillers (PP-HNT, PP-CNT and PP-CNTF) and neat PP for comparison.

#### 2.7.2. TEM 

TEM analysis was performed in a JEOL model JEM-2200FS+Cs microscope (Tokyo, Japan) with an acceleration voltage of 200 kV. Different micrographies were taken at diverse magnifications in scanning mode (STEM), transmission (TEM), electron diffraction patterns (PD) and elemental analysis (EDS). The samples analyzed by STEM/TEM include those with organosilane filler: PP-Acetic, PP-Formic and PP-Base.

### 2.8. Gas Barrier Testing

Oxygen transmission rate (OTR) of films was measured according to ASTM D3985 using a MOCON OX-TRAN® 2/21 (Minneapolis, MN, USA) gas permeation instrument based on the nanometric testing principle. In the nanometric testing method, a pressure difference (driving force) across the sample is created by maintaining the test gas at atmospheric pressure in the upper chamber, while vacuum is applied in the lower measuring chamber. While the gas permeates through the sample, the pressure in the lower measuring chamber increases. The instrument measures the time required for the lower chamber pressure to increase from a predefined lower limit to a pre-defined upper limit. The measured time interval is then transformed into the gas permeability rate expressed in mL/m^2^/day. Gas permeability of the films was determined at constant temperature (23 °C) and relative humidity (0% RH) conditions with 5–10 cm^3^/min gas flow. Permeability tests were carried out in polygonal specimens of 100 cm^2^ with an approximate thickness of 1 mm. Values reported represent average of three measurements.

### 2.9. Tensile Testing

To compare mechanical properties of samples, tests were performed in an INSTRON 3365 tensile test machine (Norwood, MA, USA) at a strain rate of 6 mm/min in accordance to ASTM 882 [15]. Tensile properties were measured on 27 rectangular specimens with a length of 10 mm, a width of 5 mm and a thickness of 1 mm. Values reported represent average from 5 measurements and typical stress–strain curves were selected for presentation in the graphs.

### 2.10. Electrical Properties

Conductivity of samples was measured using a computer-controlled system including a Keithley 6514 electrometer (Cleveland, OH, USA). For the electrical characterization *I*–*V* measurements were performed on samples of 10 × 0.7 × 0.01 cm (length, width and thickness respectively). A direct voltage *U* = *IV* was applied directly on the sample. The intensity *I* through the samples thickness *e* and contact area *S* was measured. The DC conductivity was then calculated using the following formula reported by Allaoui *et al*. [16]:

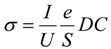
(1)

## 3. Results and Discussion

### 3.1. Molecular Weight Distribution Analysis (MWD)

The molecular weight and structure of a polymer influences the processing behavior and the physical properties of the finished product are essential for good material or product performance. Some properties that are dependent on the molecular weight are the melt viscosity, the tensile strength, toughness or impact strength, thermal resistance and corrosive properties.

**Figure 1:**
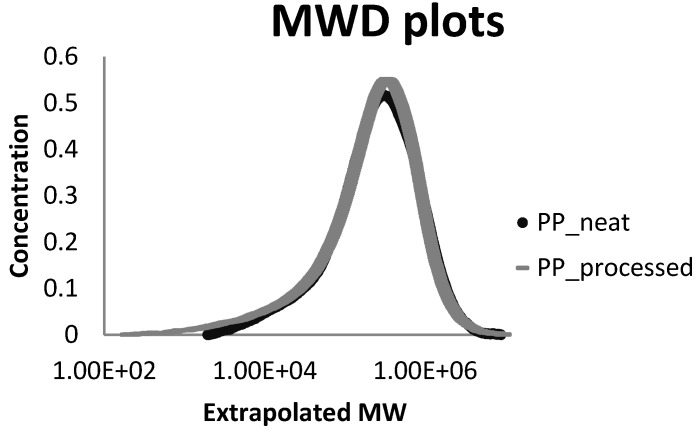
Molecular weight distribution curves as obtained from GPC measurements for the PP resins.

Table 3 presents the moments and polydispersity indices of the molecular weight distribution parameters for all the resins, and Figure 1 is the characteristic curve for both polypropylenes, as received and processed. The objective of this comparison is to ensure consistency so any change in the molecular weight distribution of the processed PP when compared to the original PP (as received) exists. If the average molecular weight in number (M_n_) is considered, the expected behavior of both polymers should be similar. Most thermodynamic measurements are based on the number of molecules and hence depend on M_n_ (e.g. colligative properties). The same tendency is observed for the average molecular weight in weight (M_w_) and the third and fourth moments as well. They present a percentage difference of 2%, 9% and 2% respectively. With this simple analysis it can be proved that received PP is not affected when it is processed and it will not impact at all in the further results obtained. Small differences in molecular weight between the PP as received and the PP processed could be attributed to structural changes but we consider this change as non-significant.

**Table 3:** Moments of the MWD and polydispersity indices for the PP resins before and after processing.

Sample	Mn	Mw	Mz	Mz + 1	PDI
PP_neat	85,776	449,894	1,116,141	2,127,264	5.25
PP_processed	75,408	460,610	1,013,190	2,084,280	6.11

### 3.2. Thermal Analysis

**Figure 2:**
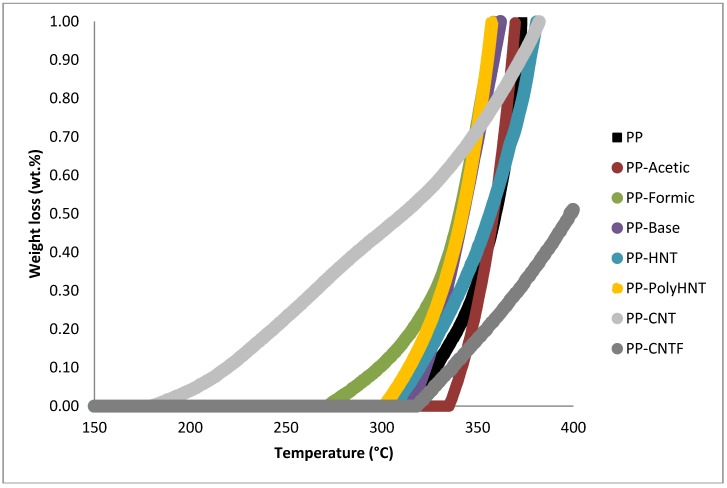
Temperature range where samples start the weight loss (degradation starts).

**Figure 3:**
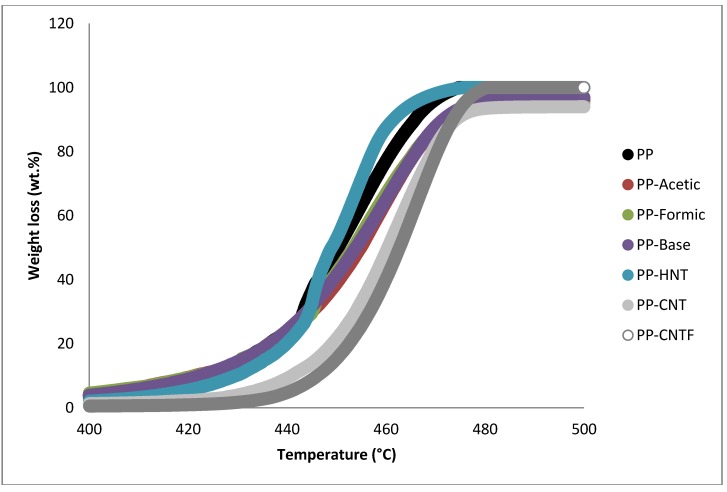
Temperature range from 400 °C to 500 °C where the higher weight is lost.

TGA curves for the samples in nitrogen are shown in Figure 2 and Figure 3 and the characteristic weight loss temperatures are summarized in Table 4. The most notorious change in weight loss is presented in the range of 440–460 °C although significant loss in mass starts around 400 °C. The first range of temperature reveals that PP-CNT followed for PP-Formic are the two samples that start degrading first. In the second and third stage it can be observed that the weight-loss percentage remain similar for almost all samples but contrary to the first stage there is a decrease in the degradation rate of samples containing CNT. The range 440–460 °C confirm that the lower degradation rate belongs to samples containing CNT (untreated and functionalized), followed by samples containing silanols and resulting the worst performance for samples containing HNT. PP-CNT and PP-CNTF continue degrading in the range of 460 °C to 550 °C and here also have their higher losses. PP containing functionalized HNT is already fully degraded in this range of temperatures and it is possible to observe that the 3 wt% of inorganic compounds is remaining making it more stable.

**Table 4:** Weight loss divided in temperature ranges where changes in weight derivate were observed.

Sample	Weight loss %				
	175–325 °C	400–420 °C	420–440 °C	440–460 °C	460–550 °C
PP	0.07	5.13	15.35	53.30	22.78
PP-Acetic	0.00	5.10	13.52	41.42	32.05
PP-Formic	0.27	4.84	13.61	43.99	28.39
PP-Base	0.14	5.06	15.29	40.90	32.23
PP-HNT	0.15	3.49	14.70	67.22	12.57
PP-PolyHNT	0.21	5.11	14.61	64.35	3.44
PP-CNT	0.56	0.96	6.96	39.84	45.04
PP-CNTF	0.04	0.39	3.85	35.61	59.63

Previous investigations suggested that the barrier properties of the nanoscale fillers were responsible for the enhancement of thermal stability of nanocomposites [3]. It is appreciated in the range of 420 °C to 460 °C that the degradation rate of samples when compared with pure PP was impacted positively. Gilman believes that the barrier properties could include both the thermal barrier, which protects the polymer from contacting with fire, and the mass transport barrier, which slows down the escape of volatile products during the process of degradation [17,18].

It can also be noticed that most of the samples start degrading before PP by itself but then the slope of degradation decreases for all samples when compared to pure PP.

Therefore it could be concluded that the barrier effects of HNTs is not the leading factor in determining the thermal stability of PP/HNTs nanocomposites. Some other investigations indicate that the iron oxides in the silicate fillers and carbon nanotubes could act as flame retardant additives and lead to some radical trapping during the process of degradation, thus enhancing the thermal stability of nanocomposites [19,20].

**Figure 4:**
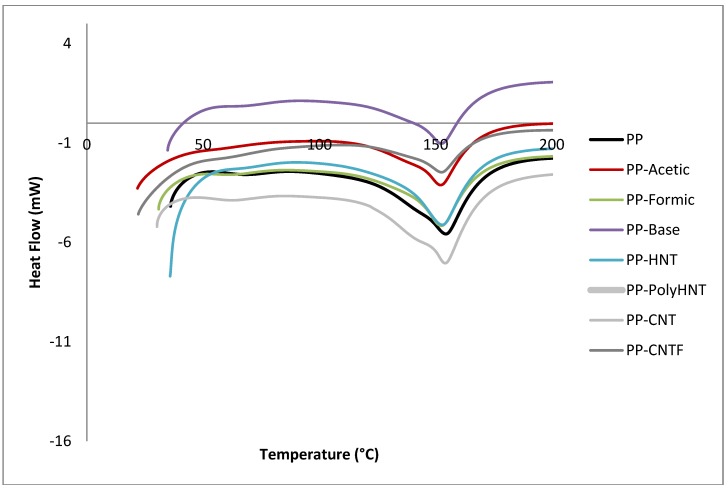
DSC curves extracted from DTA analysis for all samples.

Table 5 summarizes the results presented in Figure 4. No significant change is observed comparing the melting temperatures with respect to the pure PP with exception of the composite containing the functionalized HNT’s which melting point is reduced in 8 units compared with pure PP and 7 units compared with untreated HNT’s. The appearance of a second peak around 450° could be the result of ceiling temperature where the polymer starts to revert to their monomers as stated by Galia *et al*. [21] or crystallization of the samples where molecules tendency is to reorganize themselves. This result can be attributed to the effect of the filler added to the samples, where these particles are acting as nucleate centers to promote crystallization [3]. It is interesting to investigate the effect of fillers with a high aspect ratio on the crystalline behavior of PP being a semi-crystalline polymer. Its main, stable crystal form is the (monoclinic) form. The orientation of crystallites under stress or in the presence of some filler with a high aspect ratio, such as magnesium hydroxide or talc, can occur. It is believed that the orientation of crystallites can improve the mechanical properties [22,23].

**Table 5:** Temperatures where first peak appear in DSC referring to melting temperature.

Sample	1^st^ Peak temperature (°C)	2^nd^ Peak temperature (°C)
PP	154.55	455.91
PP-Acetic	152.17	460.4
PP-Formic	152.49	457.96
PP-Base	152.56	461.39
PP-HNT	152.92	455.47
PP-PolyHNT	146.07	454.68
PP-CNT	154.16	464.59
PP-CNTF	152.52	467.86

### 3.3. Rheological Testing

The rheological properties of PP and its nanocomposites, with HNT, CNT, CNTF and synthetized products are represented in the following figures.

**Figure 5:**
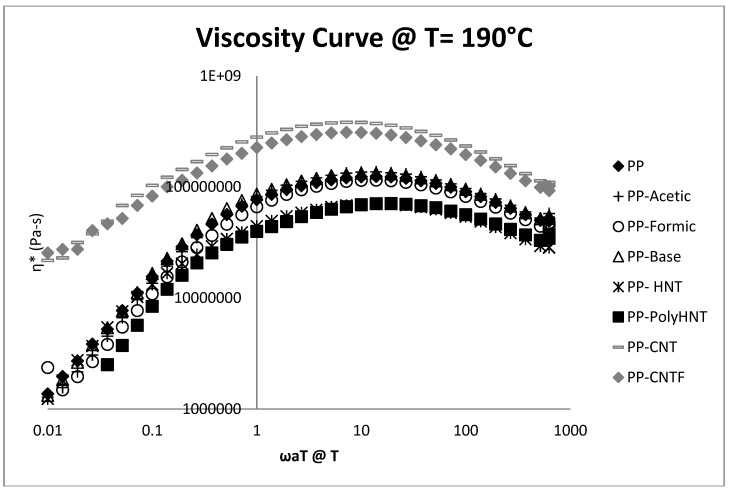
Viscosity curve @ 190 °C for all samples.

Figure 5 shows the viscosity curve of the samples at 190 °C as a function of the frequency. The results clearly indicate that viscosity of composites containing CNT untreated and modified increased when compared to pure PP. For samples containing synthetized molecules via sol-gel process no significant changes are present due to that all three samples (PP-Acetic, PP-Formic and PP-Base) overlap the Pure PP curve. For samples of PP containing treated and untreated HNT’s viscosity is reduced but not a great difference is presented in PP filled with treated or untreated HNT’s suggesting that the nanotubes treated with Poly (acrylic acid) does not create a big effect in this property.

**Figure 6:**
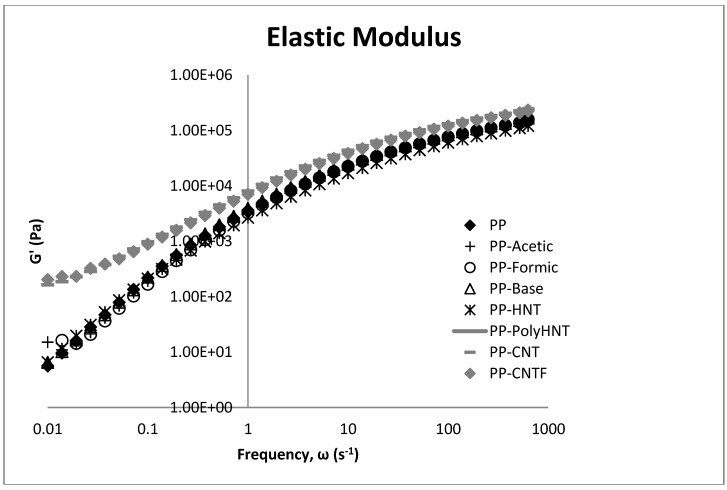
Storage modulus (G') for all samples.

**Figure 7:**
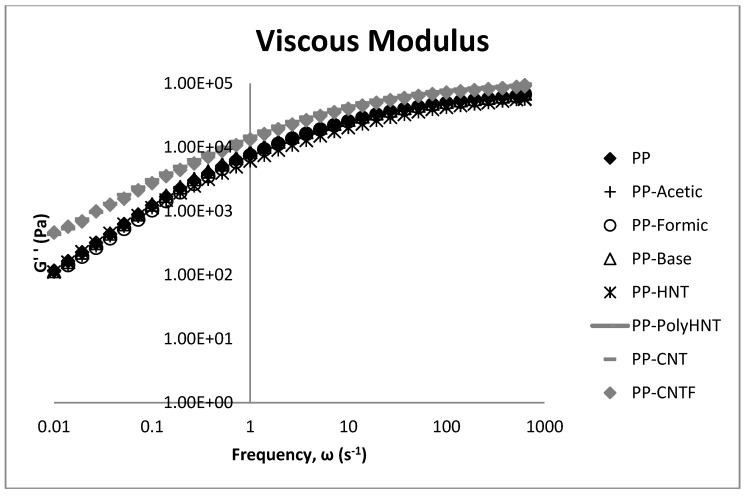
Loss modulus (G'') with frequency sweeps as a function of the filler type.

Figure 6 and Figure 7 show the elastic and viscous modulus. Storage modulus of samples increases as well for samples containing carbon nanotubes, which is due to the reinforcement effect and restrictions in the chain mobility. This behavior is not observed for samples containing HNT which would be expected due to the Silicon presence in the molecular structure. It can be assumed that this behavior could be because of a major compatibility between carbon atoms with the polymeric matrix and silicon in the HNT structure. For PP containing synthetized products the storage modulus do not present any significant change when compared to neat PP. This property resulted increased only for samples containing CNT’s this means that the incorporation of CNT into the PP matrix remarkably enhances stiffness and load bearing capability of the material.

**Figure 8:**
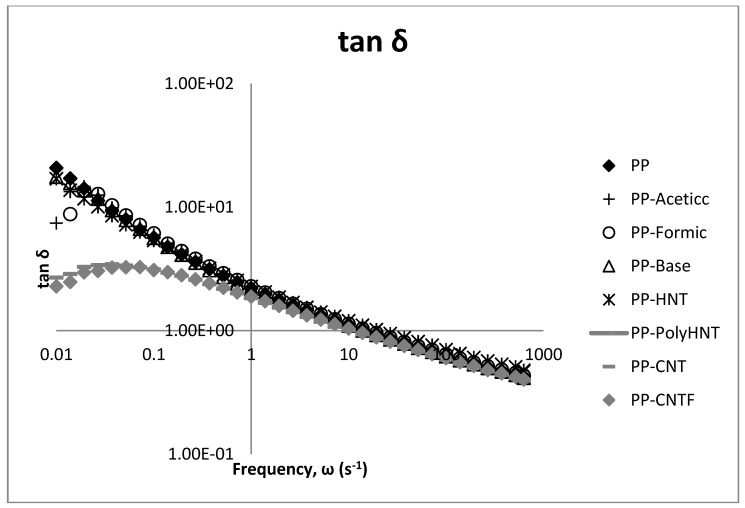
tan δ with frequency sweeps as a function of the filler type.

Figure 8 illustrates the effect of the fillers in the loss factor (tan δ) for PP composites.

In general, it can be said that the storage and loss modulus behave similar but at low frequencies the changes for CNT’s composites when compared to the others samples are better appreciated specially in tan δ plot. These analyses show a clear difference between the composites containing CNT which could be attributed to the compatibility between the additive as mentioned before (silanol or filler) and the polymeric matrix, and for these results we can say that the processability of samples containing silanols synthetized in any media and samples containing HNT is similar to the processability of neat PP. Contrary to this behavior, samples containing untreated and functionalized CNT significantly when compared to all the other samples. Both modulus resulted significant increased.

### 3.4. Morphology

Scanning electron microscopy and transmission electron microscopic analysis were conducted on cryofractured nanocomposite samples in order to investigate the nanotube dispersion and interfacial features in nanocomposites.

#### 3.4.1. SEM

**Figure 9:**
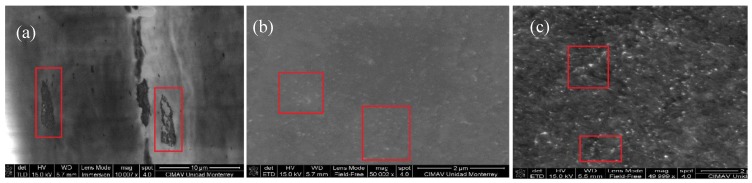
SEM images for samples (**a**) PP-HNT; (**b**) PP-CNT and (**c**) PP-CNTF. Upper images show the morphologies at ×100,000 and lower images in immersion mode taken at ×200,000.

PP-HNT (Figure 9a) does not present the same morphology than the samples containing carbon nanotubes, although it contains as well some micrometric agglomerates containing halloysite nanotubes and the fibrous structure of this material was successfully detected by the microscope. The following figure (Figure 9b) presents the sample containing carbon nanotubes but with no treatment on the surface. This sample presents agglomerates in micrometric scale but it seems to be homogeneously distributed along the analyzed surface. Finally, the polypropylene containing the functionalized carbon nanotubes (PP-CNTF) is presented and also brighter spots homogeneously distributed along the surface are appreciated. In Figure 9a it can be appreciated that probably the agglomeration of HNT caused accumulation of stresses that eventually promote the rupture of the film, these zones were not observed in the neat PP sample. The analysis at higher magnification showed that these zones are agglomerates of carbon nanotubes in micrometric scale. These agglomerates were shown dispersed in all cuts/slices analyzed.

#### 3.4.2. STEM/TEM

**Figure 10:**
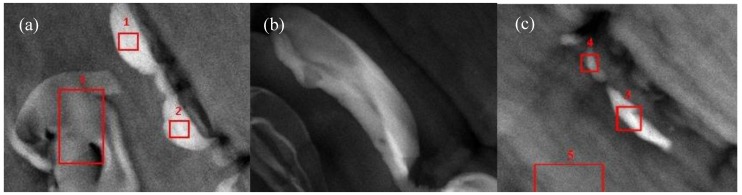
STEM images for (**a**) PP-acetic; (**b**) PP-formic and (**c**) PP-Base samples. Brighter locations show the presence of silicon molecules.

Figure 10 was obtained via STEM and they contain a clear field and a Z-contrast where the atomic mass is detected. This analysis is more oriented at the detection of elements present in each sample.

Silicon was detected in the zones analyzed and where only carbon was found refers to the composition of the PP matrix. With this information one can suppose that the formation of agglomerates of the synthetized products in these samples were found. But one important thing on doing this test was to prove the presence of Silicon as the molecular filler assembled to the PP matrix, which impact in properties will be compared to the effect of having tubular filler. In general, it can be said that all the samples presented an intercalated dispersion of filler as supported by the micrographies already described above.

### 3.5. Gas Barrier Properties

Nanoparticles are well known to reduce the oxygen permeability due to the barrier effect produced by fillers building a tortuous path in polymeric matrices. In the case of PP/MWCNT the studies of gas permeability are limited as well for HNT. For samples containing silanols as fillers literature is even more limited. Because of the same behavior in rheological properties in samples of PP containing the synthetized products, oxygen permeability test were carried out just for the sample containing silanols using formic acid as a catalyst. Thus, it is believed that their addition in the resin matrix would enhance its barrier properties by forcing the gas molecules to follow a more tortuous path as they diffuse through the material, retarding the progress of the phenomenon. This has been observed in many polymer/layered silicate nanocomposites as well as in PP using fumed silica nanoparticles [24,25].

Table 6 lists the oxygen transmission rate of samples. We mentioned before that similar conducts were observed for samples of PP containing synthetized organosilanes oxygen transmission rate was measured randomly only for one of those samples. It is believed that the presence of fillers in the studied nanocomposites create a labyrinth, complicating the path that the gas molecules must follow in order to pass through the whole width of the film as reported by Bikiatis *et al*. [26]. Although it is observed that the oxygen transmission rate is not improved when compared to the pure PP for samples containing synthetized organosilanes neither for samples containing treated and untreated CNT’s. One reason for this behavior is that fillers could form clusters indicating that a good dispersion for these additives was not completely achieved or compatibility between the filler and the polymeric matrix was not achieved. Although a remarkable improvement is observed for samples containing HNT, where OTR was reduced significantly. Similar behavior was achieved in samples containing treated HNT´s what can make us corroborate the effect of these natural nanotubes in the improvement of the barrier properties.

**Table 6:** Oxygen transmission rate of samples in (cc/m^2^-day).

Sample	Cell A	Cell B	Average
PP	404.61	420.50	412.56
PP-Formic	662.59	823.96	743.28
PP-HNT	125.79	151.90	138.85
PP-PolyHNT	90.26	84.94	87.60
PP-CNT	452.16	583.76	517.96
PP-CNTF	6312.96	13703.50	10008.23

### 3.6. Tensile Testing

Table 7 lists the tensile properties of the PP and PP composites. It can be observed that the maximum stress increases for almost all samples excluding PP containing the silanol obtained in formic acid for which the stress decreased. The maximum stress was achieved for the PP containing silanol synthetized in acetic acid followed by CNTF. The maximum strain was reached by the same samples. This property increase should be attributed to the changes in crystal structure and orientation of PP induced by the incorporation of the different fillers. Manchado *et al* [23]. reports that the physical properties of polymeric materials strongly depend on their microstructure and crystallinity. Also, he points that the modulus and toughness are the mechanical properties that are more influenced by crystallinity since failure of the material takes place at this microscopic level [23]. This fundament is also supported by Lozano *et al*. who studied thermoplastic composites reinforce with carbon nanofiber and conclude that composite strength was limited by the enhanced crystallization of the polymer brought on by nanofiber interaction as additional nucleation sites that could happen in the other samples filled with silanols and HNT [27]. As expected, the presence of CNT’s untreated and functionalized as well as PP containing a similar structure than CNTs, in this case HNT, improved the Young’s modulus. For samples containing silanol groups the higher Young modulus is presented in PP containing organosilanes obtained by sol-gel process using formic acid as a catalyst. As mentioned previously, in order to obtain better properties, a better dispersion of the fillers, a reduced number of defects, curvatures and entanglements, and enhanced adhesion between the PP and the filler is required.

**Table 7:** Mechanical properties of pure PP, PP/silanes and PP/filler composites (3 wt%).

Sample	Max stress (MPa)	Max strain (%)	Young modulus (Mpa)
PP	17.8 ± 1.4	8.6 ± 0.8	4.1 ± 0.4
PP-HNT	18.3 ± 0.4	6.8 ± 0.2	5.0 ± 0.1
PP-PolyHNT	14.3 ± 1.1	14.2 ± 0.55	2.5 ± 0.09
PP-Acetic	21.3 ± 0.9	15 ± 0.4	4.7 ± 0.2
PP-Formic	17.2 ± 1.0	7.8 ± 0.6	5.6 ± 0.3
PP-Base	17.8 ± 3.8	8.2 ± 2.0	4.0 ± 0.5
PP-CNT	19.0 ± 0.4	7.8 ± 0.3	4.5 ± 0.3
PP-CNTF	20.8 ± 1.1	8.5 ± 0.1	5.0 ± 0.1

### 3.7. Electrical Measurements

**Figure 11:**
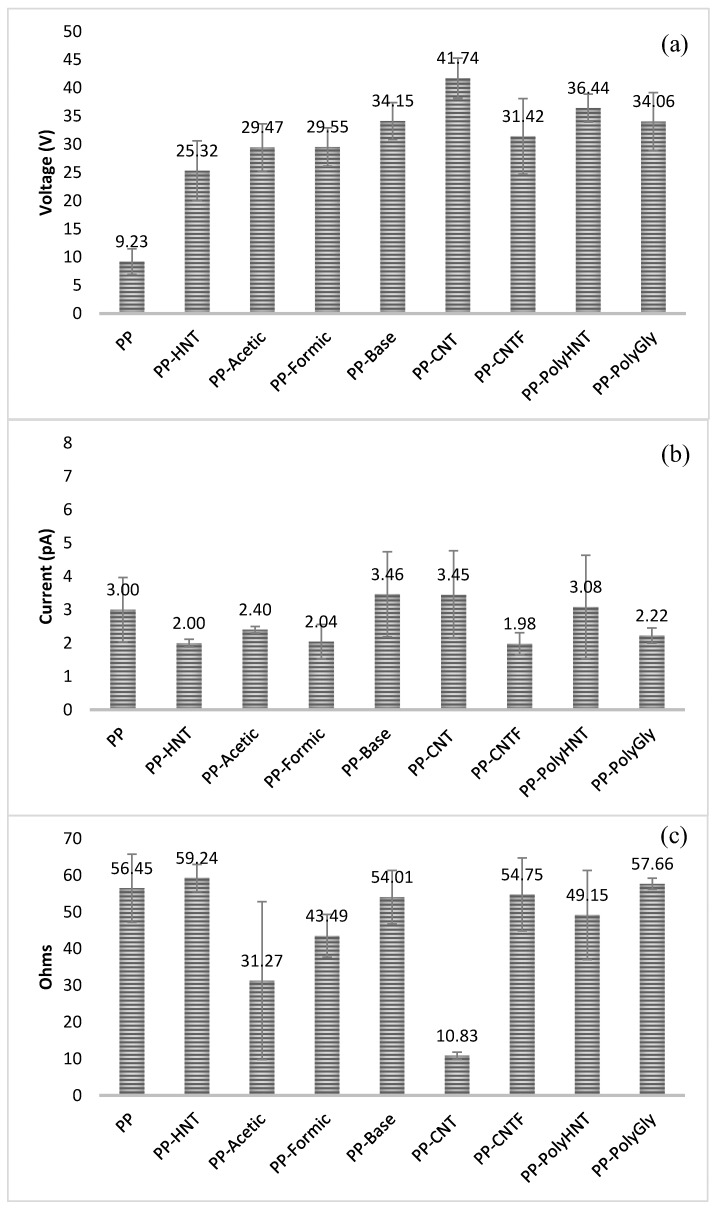
Voltage (**a**), Current (**b**) and electrical resistance (**c**) used to calculate the conductivity of samples.

**Figure 12:**
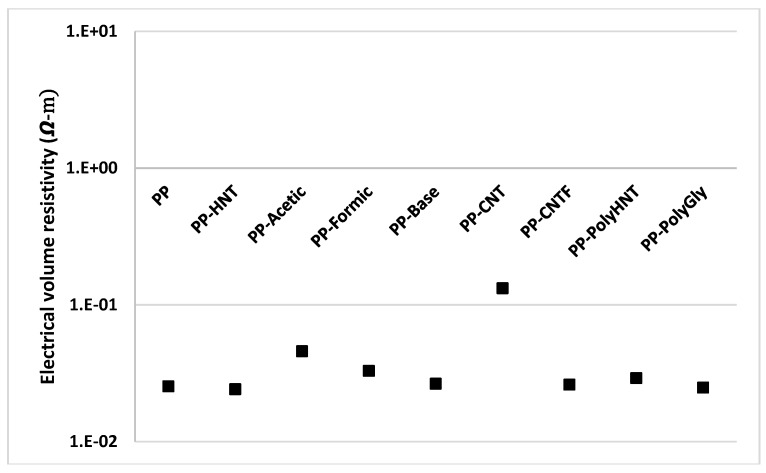
Electrical volume resistivity for all samples.

In order to analyze how electrical properties were impacted by the addition of different fillers, electrical measurements were performed and the results are presented in Figure 11 and Figure 12.

When comparing composites containing untreated CNT with the other composites containing treated CNT, HNT and synthetized silanols it is clearly seen that the electrical conductivity was found lower. Resistivity of samples resulted more or less in the same order for all samples but it is visible a significant change for samples containing untreated CNT. When comparing these samples with the functionalized CNT (PP-CNTF) this behavior could be explained due that double bonds of CNT structure were broken in order to functionalize the nanotubes. Sánchez-Fernández explanation says that there are two typical structures for O_2_ adsorption on CNT. One corresponds to physisorption at the double vacancy (type-I structure) and the other corresponds to chemical bonding with the carbon atoms (*i.e.*, chemisorption, type-II structure), which is equivalent to replacing two carbon atoms in the perfect CNT with the two oxygen atoms. The physisorption has only minor effects on the transport while the chemisorption can improve it and the resulting conductance is affected by the orientation of the O_2_ bonding [11]. In this study any catalyzer was used as Pötschke *et al* [22]. did in order to afford the *in situ* polymerization. In their study the electrical percolation was achieved at lower nanotube contents as compared with direct incorporation as did in this research.

## 4. Conclusions

To improve the load transfer in composites between the matrix and filler a covalent linking between the two components is desirable. Micrographics confirm that melt-compounding grants fairly homogeneous dispersion of the HNTs within the polymer matrix with occasionally micron-sized aggregates scattered within a matrix of neat polypropylene. Synthetized organosilanes and nanotubes (halloysite) filled polypropylene composites were successfully prepared by an industrially benign masterbatch dilution technique using high shear extrusion process. In this study the effects of three different catalyst in the sol-gel process (acetic acid, formic acid and ammonium hydroxide) has revealed that no difference in properties occurred when comparing the three products synthetized. For all characterization in samples containing PP and organosilanes synthetized in different media the properties behave similar (e.g. rheological, barrier, tensile). So we conclude that there is no impact in the catalyzers used to obtain these products; at the end the effect over the polymeric matrix will result the same. In OTR tests the samples containing HNT (treated and untreated) resulted in improving significantly the gas barrier properties when compared to neat PP whereas for other samples this property was not enhanced. Tensile properties when talking about Young’s modulus was improved for the majority of samples. To increase the compatibility between PP and HNTs, grafting PP chains onto the surface of HNTs is being carried out to increase the mechanical performance of nanocomposites. Halloysite nanotubes are unique materials and due that improvements in barrier properties were significant, its use for packaging or biomedical applications could be enormous. One of the authors in this paper, Sánchez-Fernández A. has reported and studied the effect of halloysite in *in vivo* tests suggesting that untreated HNTs at lower concentrations showed to be a highly biocompatible material, however, functionalization by selected organosilanes exhibited high cytotoxicity, inducing cell death by apoptosis [28,29]. So it can be concluded that HNTs possess promising prospects in the preparation of new structural and functional materials and due to this special efforts in improving properties using this filler are needed.

## References

[1] Turcsanyi B., Pukanszky B., Tüdõs F. (1988). Composition dependence of tensile yield stress in filled polymers. J. Mater. Sci. Lett..

[2] Liu J., Gao Y., Cao D., Zhang L., Guo Z. (2011). Nanoparticle dispersion and aggregation in polymer nanocomposites: insights from molecular dynamics simulation. Langmuir.

[3] Monsiváis-Barrón A.J., Bonilla-Rios J., Ramos de Valle L.F., Palacios E. (2013). Oxygen permeation properties of HDPE-layered silicate nanocomposites. Polym. Bull..

[4] Lozano T., Lafleur P.G., Grmela M. (2002). A chemical model for the dispersion of fillers in a polymeric matrix. Can. J. Chem. Eng..

[5] Kasaliwal G.R., Göldel A., Pötschke P., Heinrich G. (2011). Influences of polymer matrix melt viscosity and molecular weight on MWCNT agglomerate dispersion. Polymer.

[6] Savadori A., Scapin M., Walter R. (1996). Particle filled polyolefins with high stiffness and toughness, as used for load bearing components. Macromol. Symp..

[7] Li J.X., Silverstein M., Hiltner A., Baer E. (1994). The ductile-to-quasi-brittle transition of particulate-filled thermoplastic polyester. J. Appl. Polym. Sci..

[8] Ess J.W., Hornsby P.R. (1987). Twin-screw extrusion compounding of mineral filled thermoplastics: Dispersive mixing effects. Plast. Rubber Process. Appl..

[9] Wang Y., Huang J.S. (1996). Single screw extrusion compounding of particulate filled thermoplastics: State of dispersion and its influence on impact properties. J. Appl. Polym. Sci..

[10] Rong M.Z., Zhang M.Q., Zheng Y.X., Zeng H.M., Walter R., Friedrich K. (2001). Structure–property relationships of irradiation grafted nano-inorganic particle filled polypropylene composites. Polymer.

[11] Sánchez A., Cué Sampedro R., Peña-Parás L., Palacios-Aguilar E. (2013). Functionalization of carbon nanotubes and polymer compatibility studies. J. Mater. Sci. Res..

[12] Bonilla-Rios J. (1996). Effects of Peroxide on Molecular Weight and Rheological Properties of Polypropylene Resins.

[13] American Society for Testing and Materials (2012). Standard Test Method for Melting and Crystallization Temperatures by Thermal Analysis. ASTM E794-06(2012).

[14] Sinha Ray S., Yamada K., Okamoto M., Ueda K. (2003). New polylactide-layered silicate nanocomposites. 2. Concurrent improvements of material properties, biodegradability and melt rheology. Polymer.

[15] American Society for Testing and Materials (2012). Standard Test Method for Tensile Properties of Thin Plastic Sheeting. ASTM D882-12.

[16] Allaoui A. (2002). Mechanical and electrical properties of a MWNT/epoxy composite. Compos. Sci. Technol..

[17] Gilman J. (1999). Flammability and thermal stability studies of polymer layered-silicate (clay) nanocomposites. Appl. Clay Sci..

[18] Gilman J.W., Jackson C.L., Morgan A.B., Harris R. (2000). Flammability properties of polymer-layered-silicate nanocomposites. Polypropylene and polystyrene nanocomposites. Chem. Mater..

[19] Kashiwagi T., Grulke E., Hilding J., Harris R., Awad W., Douglas J. (2002). Thermal degradation and flammability properties of poly(propylene)/carbon nanotube composites. Macromolecules.

[20] Zhu J., Uhl F.M., Morgan A.B., Wilkie C.A. (2001). Studies on the Mechanism by which the formation of nanocomposites enhances thermal stability. Chem. Mater..

[21] Galia A., De Gregorio R., Spadaro G., Scialdone O., Filardo G. (2004). Grafting of maleic anhydride onto isotactic polypropylene in the presence of supercritical carbon dioxide as a solvent and swelling fluid. Macromolecules.

[22] Pötschke P., Pegel S., Claes M., Bonduel D. (2008). A novel strategy to incorporate carbon nanotubes into thermoplastic matrices. Macromol. Rapid Commun..

[23] Manchado M.A.L., Valentini L., Biagiotti J., Kenny J.M. (2005). Thermal and mechanical properties of single-walled carbon nanotubes–polypropylene composites prepared by melt processing. Carbon.

[24] Vladimirov V., Betchev C., Vassiliou A., Papageorgiou G., Bikiaris D. (2006). Dynamic mechanical and morphological studies of isotactic polypropylene/fumed silica nanocomposites with enhanced gas barrier properties. Compos. Sci. Technol..

[25] Vassiliou A., Bikiaris D., Pavlidou E. (2007). Optimizing melt-processing conditions for the preparation of ipp/fumed silica nanocomposites: Morphology, mechanical and gas permeability properties. Macromol. React. Eng..

[26] Bikiaris D. (2010). Microstructure and properties of polypropylene/carbon nanotube nanocomposites. Materials.

[27] Lozano K., Barrera E.V. (2001). Nanofiber-reinforced thermoplastic composites. I. Thermoanalytical and mechanical analyses. J. Appl. Polym. Sci..

[28] Sanchez-Fernández A., Peña-Parás L., Cue-Sampedro R., Tamayo R., Riojas P., Mendoza A. (2014). Synthesization, characterization, and *in vitro* evaluation of cytotoxicity of biomaterials based on halloysite nanotubes. Proceedings of the 1st International Electronic Conference Materials.

[29] Sánchez-Fernández A., Leyva-Valdés A., Falcón-García C., Mena-Arjona J. (2014). *In-vivo* tests for evaluation of the regenerative properties of a chitosan-based gel. Materials.

